# Dr. Frederic Frank Durand

**Published:** 1905-12

**Authors:** 


### Obituary.

### DR. FREDERIC FRANK DURAND.

In the sudden death of Dr. Frederic Frank Durand, at his residence in Maplewood, New Jersey, on Sunday, October 29th, 1905, the dental profession has lost another of its older practitioners, whose ranks are rapidly diminishing.

He had but recently returned from his summer home at Lake George, N. Y., apparently in the best of health, and the announcement of his sudden demise was a sad surprise to his many friends. Without warning he was stricken with heart trouble, and while conversing with his wife he succumbed immediately.

Dr. Durand was born in New York sixty-six years ago and was a son of the distinguished artist, Asher B. Durand. He was educated in private schools in that city, the preparatory school at Lee, Mass., then taking the scientific course in Civil Engineering and Architecture at Rensselaer Polytechnic Institute in Troy, N. Y.

The Civil War then coming on, and inactivity of business, decided his entering the dental profession. Dr. Edwin James

Dunning, who was a personal friend of his father, and one of the leading dentists of that day, persuaded young Durand to enter his office as a student—which at that time was known as the "Dunning School of Dentistry."

In addition to those facilities afforded under his teaching he took a course in Anatomy and Materia Medica at the College of Physicians and Surgeons, New York, completing his dental education in the Baltimore College of Dental Surgery, where he graduated in 1863.

He remained associated with Dr. Dunning until the latter retired from practice in 1874, when with Dr. George Elias Hawes, also an associate of Dr. Dunning's, under the firm name of Durand & Hawes a partnership was formed to continue the practice established by Dr. Dunning at 11 Waverly Place in 1856.

Upon Dr. Hawes's death in 1880 he was succeeded by his brother, Dr. John B. Hawes. Owing to business encroachment in the lower Fifth Avenue residential section, Durand & Hawes were compelled to leave Waverly Place, the house in which Dr. Dunning had established his practice twenty-five years before, locating at 13 West Thirty-second Street, where they remained until 1894, when conditions again required removing to their present address, 20 West Forty-seventh Street. With the death of Dr. Durand terminated a partnership of over thirty years' standing and, including the Dunning school, an established practice of half a century. Dr. Durand was in active practice over forty years and had devoted his life to the professional training of young men who were associated with him,—a worthy teacher of his predecessor's system; and a large number of dental practitioners who came in close contact with him will cherish his memory for its valued friendship and teachings. Although in his 67th year, he continued in active practice, travelling daily between his country home and New York.

Outside of his profession Dr. Durand was very much interested in art. He was one of the founders of the American Water Color Society, and devoted much of his vacations to art work, mostly landscapes.

He was also greatly interested in the welfare of his home community, where he had spent most of his life; the landed property he inherited had been in his father's family upward of one hundred and thirty years.

He was an active member of the Maplewood Improvement Association, and Chairman of the Board of Governors of its Public Building Company.

Dr. Durand was married in 1903 to Miss Helen Thompson, of Louisville, Kentucky, who survives him, also a brother, Prof. John Durand, of Paris, and one sister, Mrs. George B. Woodman, of New York.

